# Development and Effects of FTY720 Ophthalmic Solution on Corneal Allograft Survival

**DOI:** 10.1038/srep16468

**Published:** 2015-11-12

**Authors:** Zhaochuan Liu, Haotian Lin, Chulong Huang, Wan Chen, Wu Xiang, Yu Geng, Weirong Chen

**Affiliations:** 1 State Key Laboratory of Ophthalmology, Zhongshan Ophthalmic Center, Sun Yat-sen University, Guangzhou 510060, China

## Abstract

Fingolimod (FTY720), a novel class of sphingosine 1-phosphate receptor modulators, has received special interest among ophthalmologists, particularly given that oral administration of FTY720 has proven to effectively treat corneal graft rejection in animal models. However, no studies have examined the performance of FTY720 as an ophthalmic solution in reducing corneal rejection in high-risk corneal rejection models, and the stability and ocular irritation profile of FTY720 ophthalmic solution are also unknown. Thus, we developed 0.1%, 0.2% and 0.5% FTY720 ophthalmic solutions and evaluated their chemical stabilities under various storage conditions with high- performance liquid chromatography. To investigate the ocular irritancy of the FTY720 ophthalmic solution, New Zealand albino rabbits were subjected to the Draize test. Furthermore, classic, well-established rat allogenic penetrating keratoplasty models were used to investigate the anti-rejection efficacy of the tested FTY720 ophthalmic solutions. We found that the non-irritating 0.5% FTY720 ophthalmic solution could prolong corneal allograft survival in rats with significant efficacy for about one month. Furthermore, no significant concentration changes occurred in any of the types of FTY720 ophthalmic solutions within three months. These results revealed crucial profiles of FTY720 ophthalmic solutions and warrant further investigation and optimization of FTY720 in the anti-rejection therapy after keratoplasty.

Corneal diseases represent the second greatest cause of blindness globally^1^. A variety of corneal conditions, such as severe traumatic damage, ocular chemical injury, keratoconus and corneal dystrophies, sometimes require allogenic keratoplasty to save visual function. Keratoplasty, also known as corneal transplantation, is the most common surgical procedure in which a damaged or diseased cornea is replaced by a donated corneal graft; as such, keratoplasty is recognized as one of the most successful solid organ transplants^2^. However, corneal graft rejection remains the leading cause of human allograft failure^3^. Williams *et al.* reported that the probability of penetrating corneal graft survival in the whole cohort studied was 87%, 73%, 60%, and 46% at 1, 5, 10, and 15 years, respectively^4^. Moreover, in high-risk grafts that have prevascularized graft beds, the success rate drops dramatically, to as low as 20% to 40%^5^,^6^,^7^. In clinical practice, the main strategy for the management of corneal rejection is medication. Immunosuppressive drugs, such as corticosteroids, cyclosporin A (CsA) and tacrolimus (FK506), are often topically applied after allogenic keratoplasty to control the activity of the recipient’s immune system and reduce the risk of corneal rejection^8^,^9^. However, challenges exist in the management of corneal rejection. Corticosteroids have been shown to cause severe ocular diseases, such as glaucoma and cataracts, after long-term application^10^. Due to their hydrophobic characteristics and high molecular weights, calcineurin inhibitors, such as FK506 and CsA, have difficulty penetrating through the cornea and reaching a therapeutically effective intraocular level^11^. Furthermore, FK506 is unstable and can epimerize to two types of tautomers in aqueous solution^12^. Because of these shortcomings, the currently available immunosuppressants are still unsatisfactory for anti-rejection management after keratoplasty.

Fingolimod (FTY720), a sphingosine 1-phosphate receptor modulator, is a member of a new class of immunomodulators; in 2010, this drug became the first oral therapy to be approved by the FDA for treating relapsing and remitting multiple sclerosis (RR-MS)^13^,^14^,^15^. As a metabolite of the ascomycete *Isaria sinclairii*, FTY720 is remarkably potent in effectively suppressing Th1-mediated allograft rejection in various experimental animal models and in humans^16^,^17^,^18^,^19^,^20^. After it is phosphorylated *in vivo*, FTY720 acts as an agonist analog by binding to and decreasing the expression of sphingosine 1-phosphate receptor 1, which is normally required for T-cell egress from secondary lymphoid tissue; this decrease in expression causes the sequestration of lymphocytes from the peripheral circulation to the lymph nodes, thus preventing lymphocyte migration to the inflammatory sites and grafted organs^21^,^22^,^23^,^24^,^25^.

Recently, FTY720 has received special interest among ophthalmologists because oral administration of FTY720 has been shown to be effective for treating corneal graft rejection in animal models^26^,^27^,^28^,^29^. However, no studies have examined the performance of FTY720 as an ophthalmic solution in reducing corneal rejection in the high-risk corneal rejection model, and the stability and ocular irritation profile of FTY720 ophthalmic solutions are unknown.

Hence, in our study, we prepared 0.1%, 0.2% and 0.5% FTY720 ophthalmic solutions and evaluated their chemical stabilities under various storage conditions using high-performance liquid chromatography (HPLC). The Draize test, an acute toxicity test devised in 1944 by Food and Drug Administration (FDA) toxicologists John H. Draize and Jacob M. Spines, was performed in New Zealand albino rabbits to assess the ocular irritancy of the FTY720 ophthalmic solutions. Finally, the efficacy of the FTY720 ophthalmic solutions at overcoming rejection was investigated in the rat allogenic high-risk keratoplasty model.

## Results

### FTY720 Ophthalmic Solution is Stable under Various Preservation Conditions

**Figure 1:**
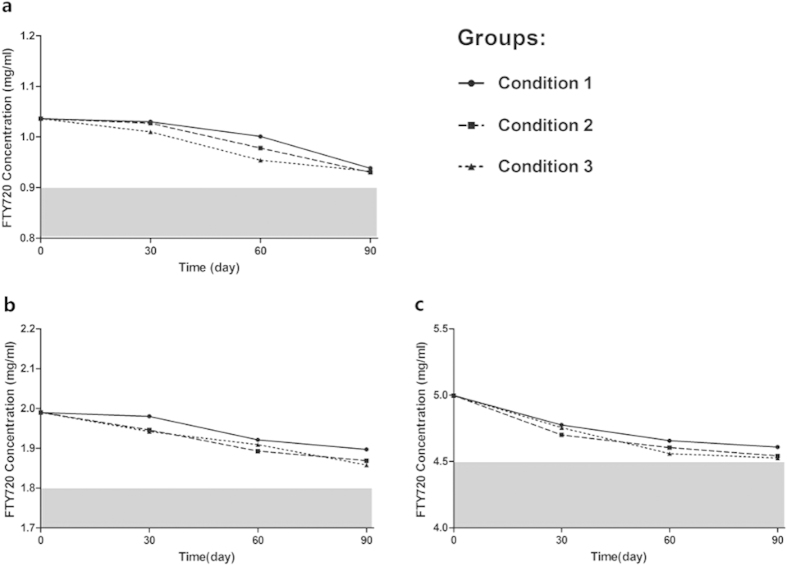
Stability of FTY720 ophthalmic solutions under three storage conditions. Condition 1: 25 °C with protection from light; Condition 2: 25 °C without protection from light; Condition 3: 40 °C with protection from light. (**a**) 0.1% FTY720 ophthalmic solution; (**b**) 0.1% FTY720 ophthalmic solution; (**c**) 0.5% FTY720 ophthalmic solution.

The results of stability assessments of the FTY720 ophthalmic solutions under various conditions are shown in Fig. 1, and the FTY720 concentrations, as determined by HPLC, can be found as Supplementary Table S1 online. Under each condition, the FTY720 concentration (in the three different formulations) was greater than 90% of the initial concentration throughout the entire storage period. All FTY720 formulations were stable under regular storage conditions (25 ± 1 °C) and when stored at higher temperatures (40 ± 1 °C) for three months. Moreover, no significant decrease in FTY720 concentration was observed when the solutions were exposed to light at the regular storage temperature. During storage, all solutions were clear, and no visible changes were observed. These results indicate that these solutions of FTY720 are acceptably chemically stable for at least 3 months, a time that is greater than the maximum follow-up of the study of FTY720 efficacy on rat keratoplasty models.

### Ocular Irritation Study of FTY720 Ophthalmic Solutions

**Figure 2:**
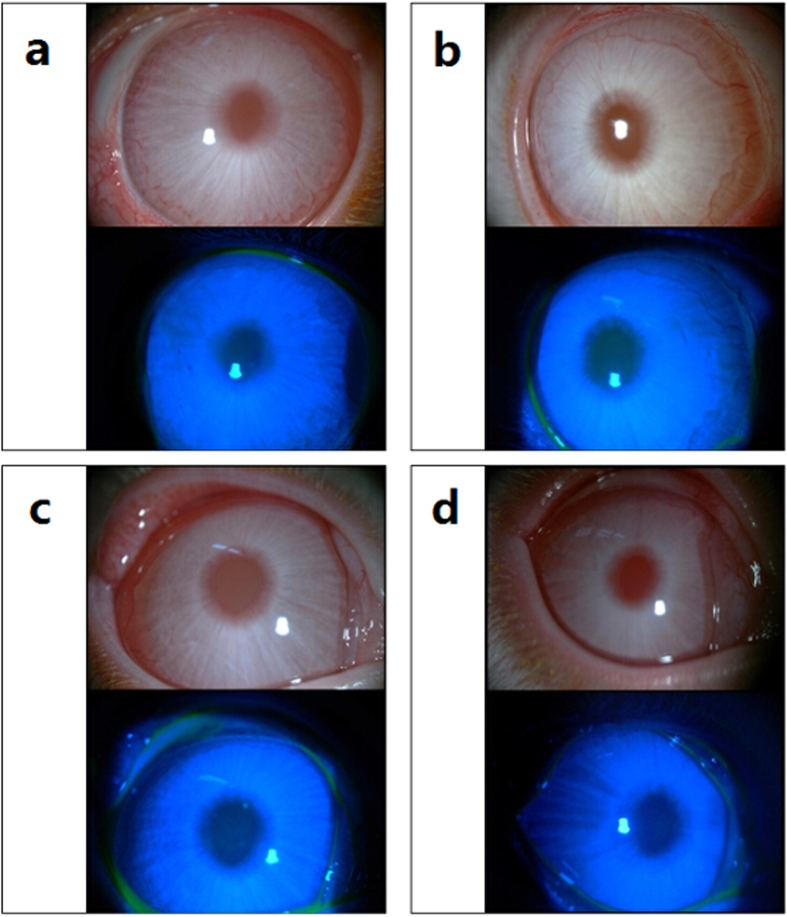
Ocular irritant scores for the FTY720 ophthalmic solutions at the end of the Draize test. Topical reactions were observed under a slit-lamp microscope after the *in vivo* instillation of normal saline or the 0.1% FTY720, 0.2% FTY720 or 0.5% FTY720 ophthalmic solution for 7 days. (**a**) Normal saline (control group); (**b**–**d**) Topical instillation of 0.1%, 0.2% or 0.5% FTY720 ophthalmic solutions, respectively.

No deaths related to the FTY720 treatment occurred, and no seizures or abnormal manifestations in animal behavior were observed during the test. The scores describing irritation of the cornea, conjunctiva, and iris were assessed, and grades for corneal and conjunctival staining were recorded. As shown in Fig. 2, no conjunctival congestion, corneal opacity or iris inflammatory exudation was observed in any of the groups. The scores of all groups met the standard of non-irritation, and no significant differences were observed between any of the three experimental groups and the saline control group. Sodium fluorescein was employed for the initial detection of breaks in the continuity and to assess the integrity of each corneal epithelium. As observed under cobalt blue light (Fig. 2), there were no ulcers, scratches or defects in the corneal epithelium when the FTY720 ophthalmic solutions were continuously administered for one week.

### Topical Use of FTY720 Ophthalmic Solution Prolongs High-Risk Corneal Graft Survival

**Figure 3:**
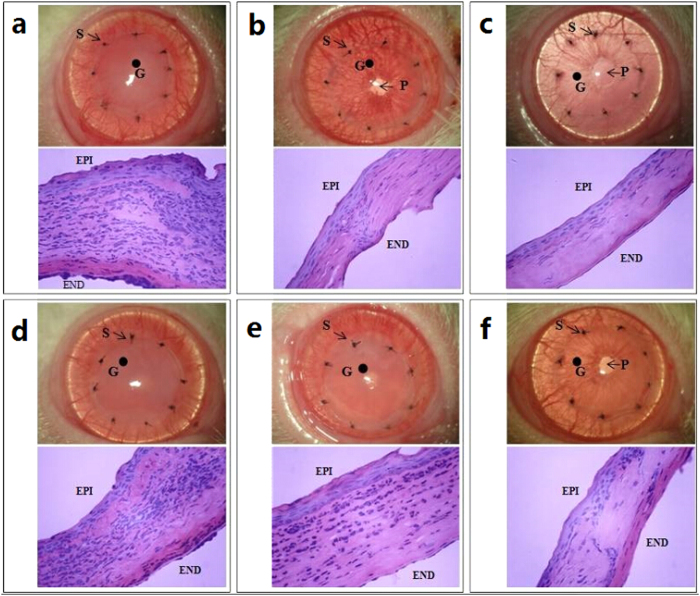
Clinical manifestations and histopathology (HE staining, magnification, 400×) of corneal grafts on postoperative day 14. (**a**) Control group; (**b**) Systemic FTY720 treatment group (1.2 mg/kg/d); (**c**) Topical instillation of 0.05% FK506 ophthalmic suspension group; (**d**-**f**) Topical instillation of 0.1%, 0.2% or 0.5% FTY720 ophthalmic solution groups, respectively. In (**a**,**d**,**e**), the pupil and iris vessels are not visible due to the severe opacity and edema of the grafts. Moreover, the degree of inflammatory cell infiltration was much greater in (**a**,**d**,**e**) than in (**b**,**c**,**f**), consistent with the clinical manifestations. S, suture knots; G, corneal grafts (the area surrounded by eight suture knots); P, pupil; EPI, epithelial layer; END, endothelial layer.

As shown in Fig. 3, the operated corneas that were treated with oral FTY720, 0.05% FK506 and 0.5% FTY720 ophthalmic solutions exhibited clear grafts on postoperative day 14. Conversely, corneal rejection, which presented as pronounced corneal opacity and corneal edema with thickening and neovascularization, was observed in the other three groups on the same day (Fig. 3a,d,e).

**Figure 4:**
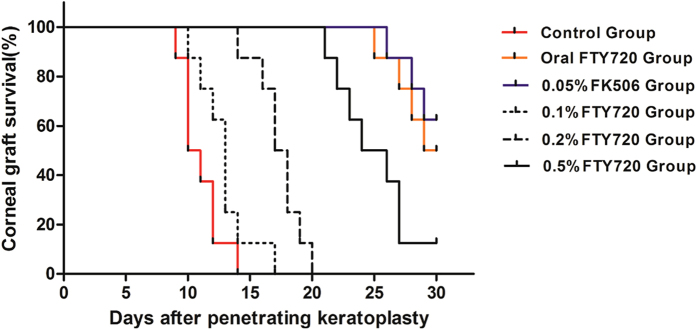
Survival curves of corneal allografts for all groups. Treatment with 0.5% FTY720, 0.05% FK506 and systemic FTY720 (1.2 mg/kg/d) significantly prolonged graft survival compared with the control condition (all p < 0.01; n = 8) or treatment with 0.1% FTY720 or 0.2% FTY720 (all p < 0.01; n = 8). Allograft survival of the control group was not significantly different from that in the 0.1% FTY720 ophthalmic solution treatment group (p = 0.08, n = 8) but was significantly different from that of the 0.2% FTY720 treatment group (p < 0.01; n = 8). Systemic treatment with FTY720 and topical treatment with 0.05% FK506 resulted in longer allograft survival than treatment with the 0.5% FTY720 ophthalmic solution (p = 0.03 and 0.01, respectively). No significant differences were observed between the results obtained after treatment with systemic FTY720 and those obtained after treatment with topical 0.05% FK506 (p = 0.59; n = 8).

The mean survival time (MST) and other relevant data are summarized in Table 1. The corneal rejection rate in the control group reached 100% within an MST of 11.0 ± 1.6 days; this rate did not significantly differ from those obtained using the 0.1% FTY720 ophthalmic solution (MST 12.9 ± 2.1 days; p = 0.08; Fig. 4). Although the rejection rate was also 100% for the 0.2% FTY720 group (MST 16.9 ± 1.7 days), graft survival in this group was prolonged compared with the control group (p < 0.01). Treatment with 0.5% FTY720 (MST 25.0 ± 3.0 days), oral 1.2 mg/kg FTY720 (28.6 ± 1.8) and 0.05% FK506 (MST 29.1 ± 1.5 days) significantly prolonged the time before rejection compared with treatment with 0.2% FTY720 (all p < 0.01) and the control condition (all p < 0.01). No significant differences were observed between the oral 1.2 mg/kg FTY720 group and the 0.05% FK506 group within the 30-day follow-up (p = 0.59), and these two groups exhibited longer graft survival times than the 0.5% FTY720 group (p = 0.03 and p = 0.01, respectively).

**Figure 5:**
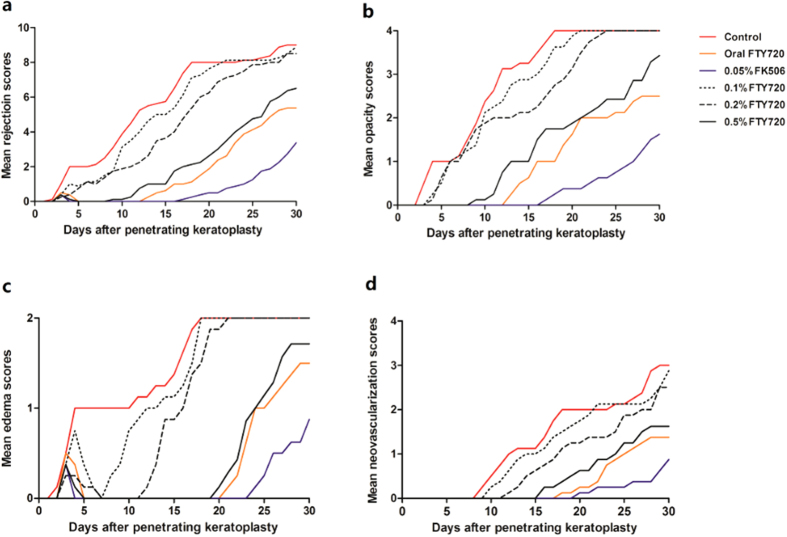
Average clinical finding scores of corneal rejection for all groups throughout the entire follow-up period. (**a**) Mean rejection scores; (**b**) Mean opacity scores; (**c**) Mean edema scores; (**d**): Mean neovascularization scores. The results obtained for the 0.5% FTY720 group, the oral FTY720 group and the 0.05% FK506 group were significantly lower than those obtained for the control group (all p < 0.05).

The average scores for total rejection, opacity, edema and neovascularization in each group throughout the entire follow-up are shown in Fig. 5. Despite the observation of transient edema in all groups within 7 postoperative days (Fig. 5c), the 0.5% FTY720 group, the oral FTY720 group and the 0.05% FK506 group presented much lower opacity, edema and neovascularization scores than the control group at other time points (Fig. 5a–d).

### Histopathological Analysis Reveals Possible Inflammation-Inhibition Effects of Topical FTY720

Histopathological analyses of enucleated formalin-fixed corneas revealed signs of graft rejection in all groups 14 days after keratoplasty (Fig. 3). The allografts in the control, 0.1% FTY720 and 0.2% FTY720 groups (Fig. 3a,d,e, respectively) revealed heavy inflammatory cell infiltration, fibroblast proliferation, edema, and significant corneal stroma thickening. Less inflammatory cell infiltration, fibroblast proliferation, and edema was observed following topical instillation of the 0.05% FK506 suspension or the 0.5% FTY720 solution or following systemic application of FTY720 (Fig. 3b,c,f, respectively) compared with the control condition. This result is consistent with the aforementioned clinical manifestations. Histopathological examination of the corneal grafts strongly confirmed the anti-rejection effects of the 0.5% FTY720 ophthalmic solution.

## Discussion

Keratoplasty is among the most widely practiced types of transplantation in humans^3^. Approximately 60,000 corneal grafts are performed every year worldwide, of which up to 30% of eyes with penetrating keratoplasty experience at least one episode of rejection, approximately 5–7% of which lead to eventual graft failure^30^,^31^,^32^. The success rate for high-risk corneal grafts is as low as 20% to 40%^5^,^6^,^7^. Therefore, anti-rejection therapy after keratoplasty is of paramount importance to save the sight of patients, especially in high-risk keratoplasty cases. However, due to their low solubility, unfavorable stability and side effects, traditional immunosuppressants are unsatisfactory for use as anti-rejection agents after keratoplasty. Thus, the development of new immunosuppressive agents has received great attention in recent years. FTY720 is a novel, high-potency immunosuppressant that is remarkably effective in a variety of autoimmune and transplant models^33^,^34^,^35^,^36^,^37^, resulting in extensive interest from ophthalmologists. Therefore, in our study, we prepared 0.1%, 0.2% and 0.5% FTY720 ophthalmic solutions and evaluated their chemical stabilities under various storage conditions with the aid of HPLC. To investigate the ocular irritancy of the FTY720 ophthalmic solutions, New Zealand albino rabbits were subjected to the Draize test. Furthermore, classic, well-established rat high-risk allogenic keratoplasty models were used to investigate the anti-rejection efficacy of the tested FTY720 ophthalmic solutions.

Systemic application of FTY720 has recently proven effective for use in corneal transplantation in animal models^27^,^28^,^29^. Sedláková *et al.* found that intraperitoneal injections of FTY720 could prolong graft survival in rat-to-mouse corneal xenografts^28^. In addition, K Mayer *et al.* reported that oral treatment with FTY720 (1.2 mg/kg/d) performed well as an anti-rejection therapy in a rat corneal transplant model^27^. In agreement with Mayer, we also found that systemic treatment with FTY720 (1.2 mg/kg/d) effectively prolonged corneal allograft survival (28.6 ± 1.8 days). However, systemic FTY720 treatment has been reported to cause hypertension, bradycardia, atrioventricular block, herpes virus infections, macular edema, skin cancer, and elevated liver enzyme levels^13^. Therefore, topical application of FTY720 is considered a useful method to avoid these adverse systemic effects. Ophthalmic solutions are the most popular drug delivery form for the management of ocular diseases, especially when these diseases occur in the anterior segment^38^. In our study, we found that the 0.5% FTY720 ophthalmic solution could effectively prolong allograft survival (25.0 ± 3.0 days) in a rat model. Furthermore, as the concentration of FTY720 was increased (from 0.1% to 0.5%), its anti-rejection effectiveness was enhanced.

Our results also demonstrated that systemic treatment with FTY720 (1.2 mg/kg/d) provided better results than treatment with a 0.5% FTY720 ophthalmic solution (p = 0.025), possibly due to low ocular drug availability. Solutions applied from an eye dropper, irrespective of the instilled volume, are often rapidly eliminated within five to six minutes after their administration, and only a small amount (1–3%) of solution actually reaches the intraocular tissue. Thus, it is difficult to provide and maintain an adequate concentration and drug retention time in the pre-corneal area. Over 75% of the applied ophthalmic solution is lost via nasolacrimal drainage, and it is also absorbed systemically via the conjunctiva; thus, ocular drug availability is low^38^. Therefore, treatment with a FTY720 ophthalmic solution, although effective, requires further improvement to enhance its efficacy by prolonging drug retention times and increasing corneal permeability^38^.

We also found that a 0.05% FK506 suspension performed better than a 0.5% FTY720 ophthalmic solution (p < 0.01). Similarly to CsA, FK506 is a calcineurin inhibitor. By binding to the immunophilin FKBP12 (FK506 binding protein), the FK506/FKBP complex inhibits calcineurin activity and then blocks Ca^2+^ -Cn-NFAT signaling, thereby inhibiting both T-lymphocyte signal transduction and IL-2 transcription, which are required for corneal allograft rejection^39^. However, the anti-rejection mechanism of FTY720 appears to be distinct from that of any other drug that has been approved or is being developed for clinical application after transplantation. FTY720 can sequester lymphocytes from the circulation to lymph nodes and Peyer’s patches, with a concomitant reduction in the recirculation of specific effector T cells from the lymph nodes to graft sites. Due to the aforementioned mechanisms, combination therapy using FTY720 with CsA or FK506 has a marked effect on prolonging allograft survival compared to monotherapy with FTY720, CsA, or FK506 alone^40^. However, Hagihara K *et al.* have argued that FTY720 might activate calcineurin signaling by stimulating Ca^2+^ influx mediated by the Yam8/Cch1 Ca^2+^ channel, an action that might counteract the synergistic anti-rejection effects of FTY720 and calcineurin inhibitors^41^. Hence, combination therapy of FTY720 with calcineurin inhibitors requires further investigation.

Stability assessment is of utmost importance in the development of ophthalmic solutions. To assess the stability of the FTY720 ophthalmic solutions, we applied HPLC, a very selective and sensitive analytical technique, to detect FTY720 concentrations under various storage conditions. Our stability assessment results indicated that FTY720 ophthalmic solutions can be stored at a regular storage temperature (25 °C) for three months. Furthermore, the results also ensured that all of the FTY720 ophthalmic solutions were stable throughout the one month of use as postoperative medication, which strongly supports the reliability of the survival analysis results. In addition to HPLC, other highly sensitive and selective analytical chemistry techniques can also be applied to detect the concentration of FTY720. For instance, N. Ferreirós *et al.* reported the application of LC-MS (liquid chromatography-mass spectrometry) to measure FTY720 in murine intracellular compartments and human plasma^42^. In addition, it is possible to apply HPLC or LC-MS to determine the ocular distribution of FTY720 after the instillation of a 0.5% FTY720 ophthalmic solution, especially in the corneal tissue and aqueous humor.

Ocular irritancy is a crucial parameter for any substance that is supposed to be administered in or around the conjunctival sac. The Draize test is a widely used classic ocular irritancy test first devised in 1944 by FDA scientists^43^. This test involves a standardized protocol according to which an agent is instilled onto the cornea and conjunctiva of laboratory animals, usually albino rabbits, and then left for a set amount of time. The effects of the agent are then evaluated. In our test, none of the FTY720 ophthalmic solutions caused signs of edema, redness, swelling, ulceration, hemorrhaging, conjunctival congestion, or opacity during the 7-day follow-up, based on the irritancy criteria. However, the Draize test is still controversial because there are anatomical and biochemical differences between rabbit and human eyes as well as ethical concerns regarding the use of animals^43^. Thus, alternative methods are required to supplement the Draize test. For instance, a human skin equivalent model and the transepicutaneous resistance test (TER) have been introduced^44^. In addition, the use of a human corneal cell line (HCE-T cells) is another good alternative method to test potential chemical-induced eye irritation^45^. Despite the disadvantages mentioned above, Wilhelmus KR *et al.* have argued that rabbit eyes are generally more susceptible to irritating substances than human eyes and that the Draize test has been successfully employed to evaluate irritant substances and prevent harm^43^.

The limitations of the present study must also be acknowledged. First, to adequately test the chemical stability of the FTY720 ophthalmic solutions, a one-year stability test under various storage conditions should be implemented. Second, because of the differences between rabbit and human eyes, the Draize test remains a controversial method to assess the ocular irritancy of chemical substances. Furthermore, although the use of a 0.5% FTY720 ophthalmic solution can prolong corneal graft survival for approximately one month, the anti-rejection effects of FTY720 over longer periods require further study. Finally, the present study did not investigate the mechanisms underlying the effects of FTY720—these mechanisms could be studied using flow cytometric analysis of T-cells and immunohistological examination of corneal grafts, for example. Despite these limitations, this is one of the first studies to reveal several key properties of FTY720 ophthalmic solutions, including their chemical stability, ocular safety and anti-rejection efficacy.

In conclusion, this study demonstrated that the non-irritating 0.5% FTY720 ophthalmic solution could prolong corneal allograft survival in a rat high-risk keratoplasty model with significant efficacy for one month and that FTY720 exhibits acceptable chemical stability for at least three months. These results warrant further investigation of FTY720-based combination therapy, the molecular mechanisms of this drug, and its ocular distribution.

## Methods

### Ethics Statement

This study and all relevant experimental protocols were approved by the Medical Ethics Committee of Zhongshan Ophthalmic Center, Sun Yat-sen University. All animal experiments were performed according to the approved guidelines established by the Animal Experimental Ethics Committee of Zhongshan Ophthalmic Center. (ethical number: 2012052)

### Preparation of Ophthalmic Solutions

FTY720 was purchased from Kangbaotai Fine Chemical Co., Ltd. (Hubei, China, batch no. 20110612, purity: 99.4%), as was its reference substance. Ophthalmic solutions of various concentrations (0.1%, 0.2% and 0.5%) of FTY720 were prepared by a senior pharmacist (Chulong Huang) according to the standard protocols^46^ of the Formulation Division of Zhongshan Ophthalmic Center (Guangzhou, China). Acetonitrile, ethanol, and trifluoroacetic acid (chromatographic grade) were purchased from Merck (Darmstadt, Germany).

HPLC was used to evaluate the concentrations of the prepared FTY720 solutions. The Agilent 1100 HPLC UV system consisted of an 1100 quaternary pump, a 100-position auto-sampler, and a variable wavelength UV-IVS detector, which was operated using the ChemStation software. Ultrapure water and methanol (both of chromatographic grade) were obtained from Honeywell International (New Jersey, USA). Chromatographic analyses were performed using a 5-μm Gemini C18 column (4.6 mm × 250 mm i.d., Phenomenex, USA). The chromatographic conditions used are listed in Table 2. The linearity, precision, accuracy, selectivity, stability, and limit of detection of this method were validated according to the guidelines of the FDA and the International Conference on Harmonization for all of the assayed matrices.

### Stability Test under Various Preservation Conditions

To assess the stability of FTY720 ophthalmic solutions, 5 mg/5 ml (0.1%), 10 mg/5 ml (0.2%), and 25 mg/5 ml (0.5%) FTY720 ophthalmic solutions were prepared and packaged in sterile vials. Three samples of each concentration were stored for up to 3 months under three conditions. Under conditions 1 and 2, the solutions (n = 3) were stored at room temperature (25 ± 1 °C). Under condition 3, the solutions (n = 3) were stored at 40 ± 1 °C in a drug stability test box (Binder KBF720, Germany). Under conditions 1 and 3, the solutions were protected from light (in colored vials). Under condition 2, the solutions were exposed to light (in colorless vials). After the solutions were stored under these conditions, they were visually inspected; then, the FTY720 concentrations of the solutions were determined using HPLC on days 0, 30, 60, and 90 of storage using the chromatographic conditions detailed above.

### Ocular Irritation Test of FTY720 Ophthalmic Solutions

The ocular irritation profiles of the FTY720 ophthalmic solutions were evaluated in New Zealand albino rabbits using the Draize eye test^43^. The eyes of each rabbit were examined using a slit-lamp microscope to ensure that the eyes were normal before performing the experiments. Nine rabbits were randomly and evenly divided into groups 1, 2 and 3, which received 0.1%, 0.2% and 0.5% FTY720 ophthalmic solutions, respectively. The FTY720 solution (0.1 ml) was administered to the right eye of each rabbit 4 times per day for 7 days, whereas normal saline solution was administered to the contralateral eye, as the untreated control, 4 times per day. Ocular responses to a single dose were observed at 1, 2, and 4 h after the first instillation. Cumulative ocular responses were observed every 24 h after the four administrations per day for 7 consecutive days. The rabbit eyes were inspected with a slit-lamp microscope at each time point, and 2% sodium fluorescein was used to detect subtle corneal epithelial damage under cobalt blue light. Ocular irritation was scored using the Draize scoring method, and irritation responses were used to score the drug as a non-irritant (scores: 0–3), slight irritant (scores: 4–8), moderate irritant (scores: 9–12) or severe irritant (scores: 13–16).

### High-Risk Keratoplasty Animal Model

All rats used in grafting experiments were females weighing 200 ± 20 g. Sprague-Dawley (SD) rats were corneal-allograft donors, and Wistar rats served as recipients. The rats were obtained from the animal care center of Sun Yat-Sen University, China. The experimental conditions used in the study conformed to good laboratory practices (National Research Council, USA, 1996), and all animals involved in this study received care in accordance with the Association for Research in Vision and Ophthalmology (ARVO) Statement on the Use of Animals in Ophthalmic and Vision Research.

Before the surgical procedures, all rats were deeply anesthetized by intraperitoneal injections of 10% chloral hydrate (2 ml/100 mg; Zhongshan Ophthalmic Center, China). Mydriasis was achieved in the eyes of the donors and recipients by local application of a tropicamide-phenylephrine ophthalmic solution (Sinqi Pharmaceutical Co., Ltd., Shenyang, China). These eye drops were administered three times at 15-minute intervals before the surgery. The donors were killed, and corneal grafts were marked with a 3.5-mm trephine and excised with Vannas scissors. Before grafting, donor buttons were stored at room temperature for approximately 20 minutes in balanced salt solution (BSS™; Alcon, Fort Worth, TX). Recipients were fixed in the left lateral position. After the right cornea was removed with a 3.0-mm trephine, the donor cornea was transplanted. The transplant was sewn in place with eight interrupted sutures (A.C.S. 10.0, Alcon, USA), which were subsequently left in place to enhance neovascularization, which leads to a high likelihood of graft rejection. At the end of each operation, the anterior chamber of each eye was restored by an injection of BSS, after which tobramycin (S.A. Alcon-Couvreur, N.V.) ointment was applied.

### Evaluating the Topical Efficacy of FTY720

After surgery, the recuperating rats were randomly divided into the groups described below (Table 3). Group 1, which served as a blank control, received the solvent without FTY720. Group 2 received oral administration of FTY720 (aqueous solution, 1.2 mg/kg/day) with a stomach tube after transplantation. Group 3 received a 0.05% FK506 suspension (0.5 mg/ml; Zhongshan Ophthalmic Center, Guangzhou, China). After transplantation, the remaining three experimental groups were treated *in situ* with ophthalmic solutions containing 0.1% (5 mg/5 ml), 0.2% (10 mg/5 ml), and 0.5% (25 mg/5 ml) FTY720 (Zhongshan Ophthalmic Center, Guangzhou, China). All of the ophthalmic agents were instilled into the grafted eyes four times per day from postoperative day 1 to postoperative day 30. In addition, all solutions were prepared at one time before keratoplasty to avoid batch bias of the FTY720 ophthalmic solutions that were applied in the rat high-risk keratoplasty model.

The rats in all groups were examined under slit-lamp microscopy every day for a maximum of 30 days. Degrees of opacity, edema and neovascularization were evaluated according to previously described criteria (see Supplementary Table S2)^47^. The day of rejection was defined as the day on which the indices of opacity, edema, and neovascularization reached moderate or severe levels, with an opacity score of ≥3 and a total score of ≥5 in grafts that were initially transparent.

Grafts with technical difficulties, such as intracameral hemorrhaging or endophthalmitis, were excluded. To enhance the accuracy of evaluation, each animal was examined and evaluated by two experienced examiners. In addition, the observers were blinded to the treatment condition. At the end of the study, the rats were killed by CO_2_ inhalation. All experimental rats were closely monitored for signs of possible toxic side effects (for example, weight loss or emaciation) throughout the follow-up.

Additionally, to perform histopathological evaluations, another 18 animals were transplanted and treated in the same way as the aforementioned groups (three rats in each group). Fourteen days after surgery, the operated eyes in each group were enucleated and fixed in 10% formalin solution. Subsequently, the formalin-fixed eyes were embedded in paraffin, sectioned into 5-μm-thick sections (using a LEICA RM2155 microtome, Germany) and subjected to hematoxylin-eosin staining for histological examination under a microscope (under a LEICA DM5000B microscope, Germany).

### Statistical Analysis

The statistical significance of survival differences among the groups was determined using the log-rank test, and the actuarial graft survival rate was analyzed using the Kaplan-Meier survival method. One-way ANOVA tests followed by multiple comparisons with least significant difference (LSD) tests were applied in all other cases. For the stability test, the average drug concentration at each time point was analyzed. P values of less than 0.05 were considered significant. Statistical analyses were performed using SPSS 19.0 (SPSS, Inc., Chicago, IL, USA).

## Additional Information

**How to cite this article**: Liu, Z. *et al.* Development and Effects of FTY720 Ophthalmic Solution on Corneal Allograft Survival. *Sci. Rep.* **5**, 16468; doi: 10.1038/srep16468 (2015).

## Supplementary Material

Supplementary Information

## Figures and Tables

**Table 1:** Mean survival time (MST) of allografts for all groups.

Group	n	Survival Days	MST ± SD (day)	Median (max/min, day)
1	8	9,10,10,10,11,12,12,14	11.0 ± 1.6	10.5 (14/9)
2	8	25,27,28,29,30,30,30,30	28.6 ± 1.8	29.5 (30/25)
3	8	26,28,29,30,30,30,30,30	29.1 ± 1.5	30 (30/26)
4	8	10,11,12,13,13,13,14,17	12.9 ± 2.1	13 (17/10)
5	8	14,16,17,17,18,18,19,20	17.4 ± 1.8	17.5 (20/14)
6	8	21,22,23,24,26,27,27,30	25.0 ± 3.0	25 (30/21)

**Table 2:** HPLC detection conditions for the studied FTY720 ophthalmic solutions.

Items	Setting
Chromatographic Column	Gemini C18 (4.6 × 250, 5 μm)
Mobile Phase (v/v)	Acetonitrile: 0.05% trifluoroacetic acid = 4:6
Detecting Wavelength	214 nm
Temperature	30 °C
Speed	1 ml/min
Time	20 min
Sample Size	20 μl

**Table 3:** Animal groups and postoperative treatment.

Group	Treatment	Medication Frequency and Time
1 (n = 8)	solvent without FTY720	4 times/d, day 1 to 30
2 (n = 8)	oral treatment using FTY720	1.2 mg/kg/d, day 1 to 30
3 (n = 8)	0.05% FK506 suspension	4 times/d, day 1 to 30
4 (n = 8)	0.1% FTY720 ophthalmic solution	4 times/d, day 1 to 30
5 (n = 8)	0.2% FTY720 ophthalmic solution	4 times/d, day 1 to 30
6 (n = 8)	0.5% FTY720 ophthalmic solution	4 times/d, day 1 to 30
