# Supplementary material for: Development and Effects of FTY720 Ophthalmic Solution on Corneal Allograft Survival
Source: Sci Rep. 2015 Nov 12;5:16468. doi: 10.1038/srep16468 (PMC4642302; doi:10.1038/srep16468)
Supplement: Supplementary Information [file srep16468-s1.pdf]

Development and Effects of FTY720 Ophthalmic Solution on Corneal Allograft Survival

Zhaochuan Liu[§], Haotian Lin[§], Chulong Huang, Wan Chen, Wu Xiang, Yu Geng, Weirong Chen[*]

Institution:

State Key Laboratory of Ophthalmology, Zhongshan Ophthalmic Center, Sun Yat-sen University, Guangzhou 510060, China.

Supplementary Tables

Supplementary Table S1. FTY720 Concentration of Each Group at Different Time Points

Groups	Storage Conditions	FTY720 Concentration (M±SD, mg/ml)			
		0d	30d	60d	90d
0.1% FTY720	1	1.036±0.048	1.030±0.015	1.001±0.009	0.938±0.029
(n=3)	2	1.036±0.048	1.027±0.020	0.978±0.028	0.930±0.023
	3	1.036±0.048	1.010±0.013	0.954±0.024	0.932±0.057
0.2% FTY720	1	1.990±0.058	1.980±0.008	1.921±0.042	1.897±2.026
(n=3)	2	1.990±0.058	1.946±0.055	1.893±0.008	1.869±0.019
	3	1.990±0.058	1.942±1.046	1.909±0.046	1.858±0.018
0.5% FTY720	1	4.998±0.087	4.776±0.085	4.657±0.066	4.609±1.039
(n=3)	2	4.998±0.087	4.700±0.080	4.605±2.047	4.542±1.073
	3	4.998±0.087	4.755±1.018	4.559±2.006	4.526±1.009

Supplementary Table S2. Scoring system used in the diagnosis of graft rejection.

Score	Clinical Findings
Opacity	
0	completely transparent
1	minimal loss of transparency
2	moderate los of transparency, but iris vessels visible
3	iris vessel not visible, but pupil outline visible
4	pupil outline not visible
Edema	
0	no edema
1	moderate edema
2	marked edema with obvious graft thickening
Vascularization	
0	No vascularization of graft
1	vessel growth to 25% of graft radius in any quadrant
2	vessel growth to 50% of graft radius
3	vessel growth to 75% of graft radius
4	vessel growth to center of graft
